# Psychometric Properties of Nursing Manager Communication Competency Questionnaire (CCCQ): An Instrument Design Study

**DOI:** 10.5334/aogh.3272

**Published:** 2021-03-12

**Authors:** Narges Asgari, Eesa Mohammadi, Anooshirvan Kazemnejad, Hassan Navipour

**Affiliations:** 1 Department of Nursing, Faculty of Medical Sciences, Tarbiat Modares University, Tehran, Iran; 2 Department of Biostatistics, Faculty of Medical Sciences, Tarbiat Modares University, Tehran, Iran

## Abstract

**Introduction:**

One of the most important factors that can enhance the efficiency and success of hospitals for achieving the objectives is the communication competency of nursing managers. Due to the lack of a local questionnaire to measure the communication competency of nursing managers, the present study has been conducted aimed to design and validate the Nursing Manager Communication Competency Questionnaire (NMCCQ).

**Method:**

First, after designing the questionnaire (production of primary items), based on the definition of the results of the study, the analysis of Schwartz, Barcott, and Kim’s hybrid concept was performed and then it was evaluated. According to the definitions of the results of Schwartz, Barcott, and Kim’s Hybrid concept analysis study, the questionnaire was designed and then it has been assessed psychometrically. Face validity with the opinion of 15 participants and content validity have been assessed using the opinion of 15 experts. Construct validity has been assessed by 300 nurses via completing the questionnaire using factor analysis.

**Results:**

First, a questionnaire with 83 items of qualitative data was made. After confirming content validity, the number of items reduced to 62 items. Exploratory factor analysis included five constructs of non-verbal communication skill, negotiation, reporting, communication ethics, and the application of communication knowledge, which consisted of 56 items that together accounted for 61.410% of the observed variance. Cronbach’s alpha coefficient for constructs ranged from 0.862 to 0.987 and the intra-class correlation coefficient (ICC) ranged from 0.969 to 0.994.

**Conclusion:**

The results showed that nursing managers’ communication competency questionnaire is a valid and reliable scale that can help managers select, assess, and appoint nursing managers. As a result, this tool can be used in future studies.

## Introduction

Although competency is a contentious issue in healthcare that is important in a variety of nursing disciplines, including nursing training, clinic, and management, it is also a complex and ambiguous concept. Although there are many definitions of this concept and several studies have been published in this field, there are many ambiguities and confusions in understanding this concept [1,2,3,4].

Rothwell (2012) defined competency as anything that results in successful work. Jackson (2003) defined competency as skills, knowledge, abilities, and other characteristics that a person needs to perform a job effectively. In a definition by Draganidis (2006), competency is a combination of explicit and implicit knowledge, behavior, and skills that empower individuals to perform effectively [5].

The psychologists refer to the term competency as a concept for measuring a person’s visible ability or performance that reflects his or her basic capacities and characteristics [6]. Competencies can be measured and expressed in the form of behavioral terms. Regardless of the professional level of preparation, the competencies required for a job are different from those of another [7,8]. Therefore, the definitions provided for competency, as well as assessment methods and competency assessment questionnaires, vary from place to place [9].

All communication interactions are to some extent situational and dependent on context and culture, and some individuals are imagined and understood as competent whose performance is consistent with the expectations and cultural context of the community. The cultural and social context is also an integral part of communication competency [10]. Nishida (1999) pointed out that individuals in a particular culture and position have a unique communication model and the main difference in communication in hospitals indicates ethnic-cultural contexts; and is racial [11]. For this reason, the questionnaire used to assess communication competency should be designed based on the same cultural and social context [10].

Regarding the complex nature of health organizations, communication of these organizations and the need for special communication competency by managers of these organizations to perform managerial tasks in the workplace, and given that communication and communication competency depends on culture and context; most studies conducted in Iran on communication competency of managers have used standard questionnaires on communication competency, such as communication skills questionnaire of Barton J. (1990) and E.C. Glenn and E.A. Pood, “Listening Self-Inventory”, which have been used in general, and some studies have used Iranian researcher-made questionnaires for managers of organizations and other professions to assess communication competency of nursing managers. Also, the questionnaire designed by Rezaei Dehaghani to assess the communication skills of nursing managers based on theoretical literature review only considers the behavioral dimension of communication competency and measures it. This questionnaire is designed from the perspective of nurses and is not suitable for self-assessment of nursing managers. Therefore, it is necessary to design a questionnaire for the communication competency of nursing managers that will assess all aspects of the communication competency of these managers and is suitable for assessing the communication competency of nursing managers from the nurses’ point of view and self-assessment of nursing managers.

## Method

The present study is a methodological one and part of a larger study with a combined method. The purpose of the study is to design and assess the Nursing Manager Communication Competency Questionnaire (NMCCQ). Designing steps were performed based on Waltz’s four-step approach (2010), including (a) selection of a conceptual model; (b) explication of objectives for the measure; (c) development of a blueprint; and (d) construction of the measure [12].

If we want to turn the definition of the concept and dimensions to appropriate and relevant items, first the theoretical definitions of each dimension were determined based on the general definition of the concept. Then, the practical definitions of each dimension were determined using the objective characteristics of each theme, and at the next stage, appropriate items were formulated to measure each of the objective or practical references as suggested.

Two qualitative and quantitative approaches were used to determine formal validity. In the qualitative method, face-to-face interviews with 15 nurses, levels of difficulty, appropriateness, and ambiguity of questions were examined and nurses were asked to express their opinions. The cases were corrected accordingly. To determine face validity quantitatively for each of the questionnaire phrases, we considered the 5-rating scale of quite important (score 5), somewhat important (score 4), moderately important (score 3), slightly important (score 2) and not important at all (score 1). If the Impact Score was equal to or greater than 1.5, the item was identified and retained as suitable for subsequent analysis [12,13].

To check content validity qualitatively, 15 professors were asked to provide their opinions after the qualitative review of the questionnaire based on the criteria of grammar, use of appropriate words, necessity, importance, and placement of items in the right place, scale, and adequacy of items.

Also, to determine content validity quantitatively, content validity index, and content validity ratio were used. For this purpose, at this stage, the questionnaire was given to 15 nursing professors who had experience in the field of scale development, and corrective comments were applied if possible. To determine the content validity index (CVI), the criterion of relevance was considered. The experts were asked to rate each of the items based on the criterion of relevance on the 4-option Likert scale. Then, at the next stage, the content validity index for each item was calculated so that the number of experts who gave scores 3 and 4 was divided by the total number of experts. Finally, a score of 0.79 and above was an acceptable score for the item. Also, to calculate the validity index of the whole questionnaire (S-CVI), the mean score of the content validity index of all items was calculated. It is acceptable if the validity index of the whole questionnaire is 0.9 or more.

To determine the content validity ratio (CVR), 15 experts were asked to express their opinion on the necessity of each item on a three-option Likert scale. To determine the minimum value of content validity ratio, according to the number of experts and based on the Lawshe Table, a score of 0.49 and above was an acceptable score for the item [14].

Construct validity helps us understand how much the tool measures the desired trait. Construct validity is sufficient to measure the existing construct. One of the methods used to determine Construct validity is factor analysis. The number of samples at this stage was five times the number of items selected.

In this study, 300 nurses from hospitals in Isfahan Province responded to the items of the Nursing Manager Communication Competency Questionnaire (NMCCQ) through a convenience sampling method. Attempts were made to sample all nurses in both genders and work with a variety of work experiences.

After collecting the completed questionnaires, the first Kaiser-Meyer-Olkin measure of sampling adequacy (KMO) and Bartlett‎ sphericity test were calculated to prove the adequacy of the number of samples used in factor analysis. At the next stage, the matrix of correlation between the variables was calculated and the factors were extracted. In this study, factor loading of 0.45 was selected and an eigenvalue was used to determine the number of factors.

Internal consistency and re-test were also used to assess tool reliability. Cronbach’s alpha coefficient was used to determine the internal consistency of each of the sub-scales and the whole tool. In this study, to determine the stability of the tool, 30 nurses were asked to complete the final tool twice every two weeks. Then, the scores of the questionnaire were calculated and the agreement between the two measurements was assessed using the index of intra-class correlation coefficient (ICC) and Pearson correlation.

This study approved by institutional review board (IRB) of Tarbiat Modares University (ethics code: IR.TMU.1394.3703 and date of approval: 7 June 2016). Before beginning the study, the consent of relevant authorities was obtained. Informed consent was obtained from participants. All procedures performed in studies involving human participants were in accordance with the ethical standards of the institutional and/or national research committee and with the 1964 Helsinki declaration and its later amendments or comparable ethical standards.

## Results

According to the designing steps of the questionnaire based on Waltz’s approach, the definition of the concept was first determined [12]. So that Nursing managers’ communication competency includes knowledge and communication skills, emotional capacity, interactive ability, and compatibility with the situation they use in their day-to-day communication with their subordinates or clients. As the communication skill of the nursing manager to communicate is compatible with the situation, for this purpose, the manager should use his emotional communication knowledge to express the ability of interactive performance using emotional capacity.

At the next stage, the objectives of the measurement are determined. In this study, the goals of the measurement are to measure the dimensions of the concept of communication competency of nursing managers, which according to the theoretical definition of the concept includes measuring communication skills, emotional competency, interactive ability, compatibility with a situation, and communication knowledge.

In the next step, map designing and estimation of the number of items were performed according to the more specific areas of communication skills, emotional competency, interactive ability, compatibility with a situation, and communication knowledge.

Fourth step: item generation and psychometry including determining face validity by the qualitative method and face-to-face interview with 15 nurses, levels of difficulty, appropriateness, and ambiguity of questions were examined and nurses were asked to express their opinions. The cases were corrected accordingly.

In quantitative face validity, three items (6, 58, and 63) did not score the item effect and could, therefore, be removed, but for review purposes, we considered opinions of respected experts and professors at content validity stage in the questionnaire and content validity of this questionnaire was confirmed with 83 items.

In qualitative content validity, based on the opinions of the respondents, changes were made to each of the items, some of which are presented in the table below. Eight items were conceptually overlapping with the other items that were removed.

According to the minimum scores of the content validity index and content validity ratio, it was decided to exclude or combine the items. Three items did not get an acceptable score for content validity index, seven items got a CVR to score less than 0.49, four items did not get acceptable CVR and CVI scores, and one item did not get any effect, CVR and CVI scores, and were excluded (***Table 1***). For calculating the content validity index based on each item, another valid index for estimating content validity is called scale content validity index (S-CVI), which is a score above 90%. S-CVI of the tool was calculated by 90.6% at this stage. Finally, 62 items entered the Construct validity stage. To determine to Construct validity five times the items, the sample size of n = 310 was determined.

**Table 1:** Face and content validity of nursing managers’ communication competency questionnaire.


ITEM	IMPACT SCORE	CVI	CVR

She talks to staff about the events and problems of the previous shift and the current situation of the department.	3.52	0.86	0.73

She talks to staff about their problems.	5	0.93	0.6

In cases where personnel are added to the workload, she talks to the physician or superior.	3.6	0.8	0.86

She tries to convince people by explaining the existing conditions.	3.52	0.93	0.6

She reminds staff when necessary.	3.76	0.73	0.73

She informs staff about what is happening in the hospital.	1.48	0.6	0.2

She explains the current situation of the department for its staff and superiors.	4.16	0.73	0.46

She explains the rules to your staff.	3.84	0.93	1

She explains to the superiors the cause of the errors.	3.44	0.8	0.6


To determine to Construct validity after collecting the completed questionnaires, the first Kaiser-Meyer-Olkin measure of sampling adequacy (KMO) and Kroit Bartlett‎ test were calculated to prove the adequacy of the number of samples used in factor analysis (***Table 2***).

**Table 2:** KMO & Kroit Bartlett tests result for the Nursing Manager Communication Competency Questionnaire’s factor analysis.


KMO index	0.956	

Kroit Bartlett test	Chi-square	12664.6

	df	1895

	Sig	0.000


The value of the KMO index is between zero and one, the closer this index is to one, the better the factor analysis. The value above 0.9 is excellent, which in this study was 0.956. This indicates that the numbers of data in this study is suitable for analysis. Also, the significance of the Bartlett‎ sphericity test suggests that sufficient correlation is between variables for factor analysis. Therefore, the factor analysis based on the correlation matrix obtained from the study sample was acceptable.

At the next stage, the matrix of correlation between the variables was calculated and the factors were extracted. Analysis of principal components is one of the most common methods of extracting factors. In this study, according to the scree plot, five factors explain the concept of the communication competency of nursing managers. The results of factor analysis showed that five factors explain a total of 61.410% of the total variance, the results of which are shown in ***Table 3***.

**Table 3:** Initial statistical characteristic of Nursing Managers Communication Competency Questionnaire.


FACTOR	INITIAL EIGENVALUES			TOTAL OF THE EXTRACTED LOADINGS BEFORE ROTATION			TOTAL OF THE EXTRACTED LOADINGS AFTER ROTATION		
		
	TOTAL	VARIANCE PERCENTAGE	CUMULATIVE VARIANCE PERCENTAGE	TOTAL	VARIANCE PERCENTAGE	CUMULATIVE VARIANCE PERCENTAGE	TOTAL	VARIANCE PERCENTAGE	CUMULATIVE VARIANCE PERCENTAGE

1	30.414	49.055	49.055	30.414	49.055	49.055	12.876	20.768	20.768

2	2.661	4.291	53.346	2.661	4.291	53.346	7.534	12.152	32.920

3	2.035	3.283	55.629	2.035	3.283	55.629	6.246	10.075	42.994

4	1.530	2.468	59.097	1.530	2.468	59.097	5.890	9.500	52.494

5	1.434	2.313	61.410	1.434	2.313	61.410	5.528	8.916	61.410


The second stage of exploratory factor analysis is factor rotation. At this stage, the factors are rotated mathematically. The purpose of performing this stage is to simplify and interpret the extracted factor construct. In this study, we used varimax rotation, which is one of the more frequent rotations with an eigenvalue above one.

After extracting the factors, each factor was named based on its items. In this study, the minimum acceptable factor load value was 0.45.

The first factor was communication knowledge with a minimum acceptable factor load of 0.486 with 21 items. The second factor was communication ethics with 13 items and a minimum acceptable factor load of 0.454. The third factor was non-verbal communication with eight items and a minimum acceptable factor load of 0.488. The fourth factor was negotiation with eight items and a minimum factor load of 0.465. The fifth factor was reporting with six items and a minimum acceptable factor load of 0.457.

To assess the reliability of the tool, we used internal consistency and retest, and to determine the internal consistency of each of the sub-scales and the whole tool, we used Cronbach’s alpha coefficient. The score range for the Cronbach’s alpha coefficient is between zero and one, with numbers closer to one indicating higher reliability of the scale. The values above 0.6 had acceptable reliability and values above 0.8 had good reliability [15,16]. In this study, Cronbach’s coefficient for the sub-scales is as follows (***Table 4***).

**Table 4:** The Cronbach’s Alpha Values for the Communication Competency of Nursing Managers Questionnaire.


FACTOR	ITEMS	INTERNAL CONSISTENCY	ICC

Reporting	6	0.862	0.994

Communication knowledge	21	0.972	0.980

Communication ethics	13	0.937	0.994

Non-verbal communication	8	0.922	0.969

Negotiation	8	0.822	0.972

Total	56	0.987	0.988


According to the results of ***Table 4***, all the factors had good internal consistency. Also, Cronbach’s alpha for the whole tool was 0.987 times, which indicates that the tool had acceptable internal consistency.

In this study, to determine the stability of the tool, 30 nurses were asked to complete the final tool twice every two weeks, then the questionnaire scores were calculated and the agreement between the two measurements was assessed using Intra-Class Correlation Coefficient Index (ICC) and Pearson’s correlation. It should be noted that ICC above 0.7 is desired [5,6,7].

The results of the above Table indicate that there is stability in all constructs of the tool. Also, the whole tool has good stability.

The Pearson test was also used to determine the stability of the tool. The correlation coefficient of 0.995 indicates that the stability of the communication competency of nursing managers is desired (***Table 5***).

**Table 5:** Pearson correlation of the Communication Competency of Nursing Managers Questionnaire.


	PEARSON CORRELATION	TEST	RETEST

Test	Pearson correlation	1	.995

	sig. (2-tailed)		.000

	N	30	30

Retest	Pearson correlation	.995	1

	sig. (2-tailed)	.000	

	N	30	30


Questionnaire scoring: All 56 final items of the tool are positive, answering items from always (5), often (4), sometimes (3), rarely (2), never (1). The highest score obtained for the questionnaire is 280 and the lowest score is 56. To determine the total score of the tool, we have drawn a histogram diagram of three, four, five, six, and 7-point Likert distributions, with the four-point distribution having the best normal distribution.

A score of 56–112 shows poor communication competency of nursing managers, a score of 113–169 shows moderate communication competency of nursing managers, a score of 170–226 shows good communication competency nursing managers and a score of 227–280 shows excellent communication competency of nursing managers.

Finally, the designed and validated questionnaire in this study was named Nursing Manager Communication Competency Questionnaire (NMCCQ). This questionnaire measures the communication competency of nursing managers with 56 items.

## Discussion

The design and psychometric process reported in the present study led to the presentation of a tool for measuring the communication competency of nursing managers, in the form of five constructs and 56 items. In the study, it was found that the tools related to the communication competence of nursing managers have not been designed so far or are mainly tools for measuring the general competence of managers and especially in Iran in many cases based on self-made questionnaires that do not have scientific design and psychoanalytic steps. To design this questionnaire, in the present study, rich knowledge of texts in international sources and contextual data were used in combination and scientific design and psychometric steps were used. The literature review showed that studies conducted in Iran and the world measured communication competency of nursing managers in many cases based on researcher-made questionnaires without the necessary validity and reliability and/or inappropriate questionnaires.

Communication Competency Questionnaire (CCQ), developed by Monge (1982), is the first tool to assess competency from an organizational perspective and can be used to assess supervisors or subordinates. However, CCQ items can be used to examine the organizational communication competencies of managers but this tool has 12 items and two factors (sub-scales), and coding and decoding skills. The sub-group of coding skills includes seven items and the decoding skills sub-scale has five items. This tool has internal reliability with an average of 0.85 for both caregivers and subordinates [17].

A comparison of constructs of CCQ (coding and decoding) with NMCCQ shows that the two questionnaires are not similar in any of the constructs, and this non-similarity can be found in the difference in the situation and conditions between groups of the questionnaires. According to the designers, CCQ is used to assess individuals who play a special role in the organization and assess communication competency of both supervisors and subordinates, while NMCCQ is specific to nursing managers, as well as NMCCQ according to the conceptual model and comprehensive definition of combining the results of concept analysis by literature review and analysis of the content of interviews has a comprehensive view of communication competency of nursing managers and examines different dimensions of communication competency of nursing managers, including the use of communication knowledge, communication ethics, negotiation skills, non-verbal communication and reporting different situations, such as the complexity of the conditions of hospitalization (workplace), shifts, adjustment difficulties and problems of shifts and interacting with the patients. While CCQ is used according to Monge to assess the communication competency of the manager as well as subordinate staff. Given that the version of the questionnaire does not differ from that of subordinate staff for assessing the manager, and none of the items in Monge questionnaire had content that reflects the relationship between managers, it seems that Monge questionnaire is not a specialized tool for assessing communication skills of managers and can be considered as a general questionnaire in the field of communication competency assessment, while in NMCCQ, there are 14 completely specialized items in the field of nursing management.

According to the full review of the items, it is important to note that Monge CCQ has only 12 items and a very brief overview of limited aspects of managers’ communication competency, while NMCCQ, with 56 items and a comprehensive view of the relationship between managers examines various aspects of communication competency such as flexibility, using different communication channels in different situations, respect, establishing appropriate communication with each person within the framework of norms, and fairness in communication which is one of the cases a nursing manager should consider with staff. Also, according to the item by item review of two questionnaires, it was found that the items in the field of communication knowledge of NMCCQ were not able to generalize or adapt to CCQ. Given that communication knowledge with 21 items as the most important construct of Nursing Manager Communication Competency Questionnaire (NMCCQ) and examines various aspects of communication competency of a nursing manager such as self-supervision, selection and use of appropriate communication method, interaction and conflict management, support, and guidance of staff, and compatibility with the situation, so it seems that in CCQ this basic dimension of managers’ communication competency has been neglected.

Accordingly, CCQ, which is a non-specific questionnaire and does not include all aspects of the communication competency of nursing managers, cannot be used to find the communication competency of nursing managers.

Communication Competency of Women Managers Scale (CCWMS) tool, designed by Berryman-Fink (1982) to measure the communication competency of women managers, includes the dimensions of interpersonal listening competency skills, verbal abilities, empathy, acceptance, non-verbal communication, and flexibility. The tool assesses communication competencies understood by the staff of women managers. It also includes items related to the training needs of women managers such as assertiveness, public speaking, credibility, professionalism, and emotional control. The scale includes 30 items, 16 positives, and 14 negative ones [18].

Nursing Manager Communication Competency Questionnaire (NMCCQ) has five constructs and 56 items. The constructs of this questionnaire include skills of non-verbal communication, negotiation, reporting, communication ethics, and application of communication knowledge. For comparing the two questionnaires, the common construct between the two questionnaires must be “non-verbal communication” which can be attributed to the fact that in most texts, one of the dimensions of communication competency is non-verbal communication [19,20,21,22].

According to the item-by-item review, it was found that the items in the field of communication ethics of NMCCQ could not be generalized or adapted to the items of CCWMS. In NMCCQ, one of the most important dimensions of communication ethics is that having 13 items is the second dimension of the communication competency of nursing managers. The structure of communication ethics also examines various aspects of communication competency such as respect, fairness in communication, motivation, and willingness to communicate, communication limit, confidentiality, calmness and self-esteem, and practical model of the nursing manager. This is one of the cases that a nursing manager should consider when communicating with his or her staff.

Communication Competency of Women Managers Scale (CCWMS) is designed for women managers only and is limited in terms of application for all managers (men and women). Given that CCWMS tool includes items related to training needs of women managers such as assertiveness, public speaking, credibility, professionalism, and emotional control, and items related to the measurement of communication competency of managers are not distinct from training needs section and the number of items each part is not clear, and on the one hand this questionnaire is designed to assess managers and is not professionally designed for nursing managers, it can be concluded that CCWMS is not a specialized tool to assess communication competency of nursing managers.

## Conclusion

According to the results of Construct validity, the concept of communication competency of nursing managers includes a set of skills of non-verbal communication, negotiation, reporting, communication ethics, and application of communication knowledge. The study results led to the presentation of the Nursing Manager Communication Competency Questionnaire (NMCCQ), which is a valid and unique questionnaire for measuring the communication competency of nursing managers. This tool is designed based on international studies along with local information and assess the communication competency of nursing managers. Using this questionnaire helps managers select, assess, and appoint nursing managers.
